# Artificial intelligence in primary care: opportunities, risks, and the road ahead

**DOI:** 10.3399/BJGPO.2025.0233

**Published:** 2025-12-19

**Authors:** Nancy Zhang, Rajnish Mohindroo, Praveen Mundlur, Rahul Mittal

**Affiliations:** 1 Rutgers University, School of Arts and Sciences, New Brunswick, USA; 2 Health and Beyond, Wolverhampton, UK; 3 Rutgers University, School of Health Professions, New Brunswick, USA

**Keywords:** Ethics, Education and standards, Artificial Intelligence, Primary healthcare

## Introduction

Artificial Intelligence (AI) is no longer a distant prospect in health care. In primary care, where the majority of patient interactions first occur, AI-driven systems are being adopted to triage symptoms, optimise workflows, and improve patient access to care. Integration of AI with online platforms and telephony systems have been evaluated to enhance patient access, satisfaction, and appointment efficiency.^1^ In parallel, AI technologies have been piloted to identify bottlenecks in service delivery, accelerate disease detection, and even automate medication dispensing through robotics.^2^ These advances are the grounds for a smarter and more accessible primary care system.

However, the development of an AI-assisted norm is accompanied by many profound dilemmas. While technological innovation advances, the legal, ethical, and professional frameworks that regulate medicine remain comparatively stagnant. A future of AI-assisted primary care cannot be realised without first addressing key concerns: a vacuum of accountability, the risk of algorithmic bias, and the erosion of trust and professional integrity in the patient–clinician relationship. This commentary argues that enforceable accountability, safeguards against bias, and protections for medical professionalism must be established before AI can be considered as a routine feature of primary care.

### An accountability vacuum

Perhaps the most pressing challenge of AI-assisted healthcare lies in accountability. Traditional medical practice follows a well-established chain of responsibility: a physician evaluates a patient, makes a decision, affirms that decision in good faith, and assumes professional responsibility for the outcome. AI interrupts this system, and this framework fractures. AI systems are not legal entities, cannot be held liable, and often operate as ‘black boxes‘ whose reasoning is inaccessible to clinicians and patients alike.^3^

This accountability gap is particularly visible in insurance and liability law. Consider a scenario in which an AI triage tool misclassifies chest pain as a non-urgent symptom. If the patient later suffers a myocardial infarction, who is responsible: the clinician who deferred to the AI, the company that developed the algorithm, or the healthcare institution that procured it? At present, no clear legal answer exists. Insurers and providers are understandably hesitant to adopt tools without defined frameworks for liability.^3^

Moreover, these implications extend beyond legalities. Accountability also underpins the trust inherent in the patient–doctor relationship. Informed consent depends on transparency: a patient consents to treatment after understanding both the physician’s rationale and the risks. However, AI systems make their decisions in a black box and provide no such transparency. Clinicians cannot interrogate the model’s reasoning, and patients cannot meaningfully consent to decisions derived from inaccessible algorithms. In this accountability vacuum, neither trust nor professional duty can be upheld.

### Algorithmic bias and digital discrimination

AI systems also possess the capacity to amplify healthcare inequities through algorithmic bias. When algorithms are trained on biased data, learned practices may become future medical procedures that are falsely believed to be objective. This results from training systems on historical data that often reflect existing disparities such as unequal access, homogenised treatment pathways, and disparate health outcomes.^4^

An AI trained with homogenised data produces standardised approaches that do not reflect the needs of a diverse community. For instance, in 2024 AI melanoma diagnostic tools performed drastically worse for darker-skinned patients.^5^ Diagnostic systems are failing to interpret cultural variations for symptom reporting and include preferred treatments. With primary care as the first intervention for disease trajectories for most patients, biases can create devastating consequences for equity in health care.^6^

The longer this issue is left unaddressed, the more difficult it will be to diagnose. The particularly insidious part of algorithmic bias is that it operates silently. Patients and practitioners may unknowingly accept biased information, misled into believing that the AI only gives objective medical judgment.

### The balance between medical integrity and technological efficiency

The third dilemma is the erosion of medical integrity in pursuit of efficiency. Primary care systems worldwide are under immense pressure: clinician shortages, rising demand, and administrative burden have made efficiency an urgent priority. AI promises relief for often overwhelmed healthcare systems by streamlining workflows, reducing documentation, and supporting diagnostic decision-making. Yet this shift risks redefining the very nature of medical professionalism.

As reliance on AI grows, clinicians risk becoming passive executors of algorithmic outputs rather than independent decision-makers. Professional autonomy, historically grounded in expertise, judgment, and accountability, may be diluted into compliance with AI-generated pathways. This is not merely a change in workflow, but also a fundamental threat to professional identity.

Clinicians, trained to make medical decisions, may find themselves reduced to the position of system navigators; instead of making judgments based on personal knowledge, they will interpret AI recommendations, amalgamate differing outputs, or attend to technological issues. Over time, this new AI-optimised workflow risks clinician burnout and erodes their identities as patient advocates.^7^

Trust is not a peripheral issue; it is foundational to effective healthcare. Patients disclose sensitive information because they trust their physician to protect confidentiality and act in their best interest. With AI in the loop, patient data which will be less private: processed by opaque systems and often owned by private entities. Concerns about privacy, data sharing, and surveillance may undermine patients’ willingness to engage fully with care.^7^

Equally important is empathy. While AI can mimic conversational patterns, it cannot replicate genuine care or compassion. For patients facing uncertainty, pain, or fear, the assurance of human empathy is irreplaceable. The danger is not only that efficiency may replace empathy, but that the value of empathy itself may be diminished in clinical practice. Preserving the relational essence of medicine is essential if AI is to serve rather than hollow out primary care.

### Call to action

AI holds an undeniable promise for transforming primary care, but this promise must be pursued with caution and responsibility. Three priorities are paramount:

1. **Establishing accountability frameworks**
  Clear legislation and professional guidelines must define liability for AI-assisted decisions. Shared responsibility should be distributed across clinicians, institutions, and developers, ensuring that patients are never left without recourse. Transparency regarding AI’s role in clinical decisions should be documented as part of informed consent.
2. **Embedding equity by design**
  AI systems must be built and deployed with equity as a non-negotiable standard. This requires representative training datasets, regular auditing for bias, and mechanisms to address inequities when identified. Primary care, as the first line of intervention, must not reproduce the structural inequities of the past.
3. **Preserving human integrity in care**
  AI should augment, not replace, clinical judgment. Systems must be designed to support human oversight, not circumvent it. Patients should have the right to understand how decisions about their care are made, and clinicians must retain the autonomy to override algorithms when necessary. Preserving empathy, trust, and professional identity is essential to the future of medicine.

## Conclusion

The future of primary care will not be judged by the sophistication of its algorithms but by the safety, trust, and equity it delivers. AI offers immense potential to enhance access and support clinical decision making. Yet without accountability, safeguards against bias, and protection of medical integrity, its integration risks undermining the very values primary care is built on.

If AI is to extend the mission of primary care, it must serve medicine’s highest ideals rather than force medicine to conform to technological limitations. The challenge for policymakers, developers, and clinicians is to craft a path where innovation strengthens, rather than erodes, the principles of equity, accountability, and trust. Only then will AI truly fulfill its promise in primary care.
